# Depressive Symptoms Among Adolescents and Young Adults Living With HIV on Antiretroviral Therapy: Outcomes From a Cross-Sectional Study in Accra, Ghana

**DOI:** 10.1155/arat/3249809

**Published:** 2025-08-20

**Authors:** Vincent Ganu, Kwasi Torpey, Margaret Lartey, Delali Fiagbe, Magdalene Akos Odikro, Veronika Shabanova, Elijah Paintsil, Ernest Kenu

**Affiliations:** ^1^Department of Medicine, Korle Bu Teaching Hospital, Accra, Ghana; ^2^Department of Population, Family & Reproductive Health, School of Public Health, University of Ghana, Accra, Ghana; ^3^Department of Medicine & Therapeutics, University of Ghana Medical School, Accra, Ghana; ^4^Department of Psychiatry, University of Ghana Medical School, Accra, Ghana; ^5^Department of Epidemiology & Disease Control, School of Public Health, University of Ghana, Accra, Ghana; ^6^Department of Pediatrics, Yale School of Medicine, Yale University, New Haven, Connecticut, USA; ^7^Department of Biostatistics, Yale School of Medicine, Yale University, New Haven, Connecticut, USA; ^8^Boston University Chobanian & Avedisian School of Medicine, Boston, Massachusetts, USA

## Abstract

**Introduction:** Systematic screening for depression among adolescents and young adults living with HIV (AYALHIV) is limited or nonexistent in some settings in sub-Saharan Africa due to the lack of integration of mental healthcare into HIV program. This can increase the risk of suicidal thoughts and cause emotional distress, which can have an adverse effect on their treatment outcomes. Establishing evidence of the burden of depression among AYALHIV is key for advocacy for systematic screening. This study examined the burden of depressive symptoms and associated factors among AYALHIV in Ghana.

**Methods:** A single-center facility-based cross-sectional study was conducted among AYALHIV at the Infectious Disease Center of the Korle Bu Teaching Hospital, Accra, Ghana, from May 2023 to March 2024. Depressive symptoms were assessed through the administration of the Patient Health Questionnaire for Adolescents (PHQ-A). The prevalence of depressive symptoms (PHQ-A score ≥ 5) and associated factors was examined using logistic regression.

**Results:** A total of 280 AYALHIV were recruited with 50.7% being female. Sixty percent were young adults, and the rest were adolescents. About 75% were virally suppressed. The prevalence of depressive symptoms was 40.7% (95% CI: 34.9%–46.7%). Being an orphan (adjusted odds ratio [aOR] = 2.6, 95% confidence interval [95% CI]: 1.2–5.6), female (aOR = 1.8, 95% CI 1.1–3.0), and having high perceived stigma (aOR = 2.2, 95% CI: 1.3–3.7) were associated with having depressive symptoms. Viral nonsuppression was associated with depressive symptoms but likely through its association with HIV stigma.

**Conclusion:** Depressive symptom burden was high among AYALHIV despite viral suppression in the majority of them. There is a need for the design of mental health screening integration into youth-centered HIV programs based on context for early identification, counseling, and treatment of mental health challenges to ensure good quality of life and avert other mental health challenges.

## 1. Introduction

Access to antiretroviral therapy (ART) has been a key intervention to the survival of children living with HIV into adolescence and young adulthood [1]. The number of adolescents and young adults living with HIV (AYALHIV) has seen a 30% increase globally between 2005 and 2016 with a significant number acquiring the infection from their mothers [2, 3]. Eighty-nine percent of AYALHIV are currently living in sub-Saharan Africa (SSA) with the majority being female [4, 5].

Globally, the estimated prevalence of mental disorders among adolescents and young adults (AYAs) ranges from 10% to 20% [6]. However, the rates in low- and middle-income countries are less established [6]. The prevalence of depressive symptoms among AYALHIV in SSA ranges from 4.4% to 52.6% [7]. Factors associated with mental health disorders among AYALHIV are female sex, older age, fewer years of schooling, and HIV-related stigma [7].

AYALHIV have to confront HIV-related psychosocial issues, inflammatory brain changes due to HIV, its treatment, and treatment adherence challenges [8]. These issues result in AYALHIV experiencing worse forms of mental disorders compared to AYAs who do not have HIV infection. These mental health disorders often go undiagnosed and untreated in AYALHIV due to a lack of adolescent-centered HIV program interventions [9, 10].

Most HIV programs are currently rolling out integrated service interventions targeted at AYALHIV based on the World Health Organization (WHO) recommendation [11]. Mental health services are one of the priority interventions for integration. Ghana's HIV program began ART in 2003 resulting in the survival of HIV infected children into adolescence. With the increasing number of AYALHIV, Ghana began to focus some of its HIV activities on this population, with the largest ART facility establishing a clinic purposely for AYALHIV for maximum attention. Ghana has also started some mental health interventions for AYALHIV but has yet to integrate mental health services into routine HIV care. Mental health disorders can adversely affect HIV treatment outcomes among this population and negatively impact their quality of life [10]. Early identification of mental health disorders among AYALHIV through screening and prompt intervention is key to improving their quality of life.

There is also a need to generate data on the existing burden of mental health disorders among AYALHIV to inform evidence-based integrated HIV programming and policy. This study sought to determine the prevalence of depressive symptoms and associated factors and their relationship to HIV care outcomes among AYALHIV in Accra, Ghana.

## 2. Materials and Methods

### 2.1. Study Design and Setting

A cross-sectional study was conducted from May 2023 to March 2024 among AYALHIV at the HIV clinic at the Infectious Disease Center of the Korle Bu Teaching Hospital in Accra, Ghana. The HIV clinic is the largest ART center in Ghana. It has over 6000 adult patients and 350 AYALHIVcurrently receiving care on an outpatient basis. All AYALHIV accessing care at the clinic have had their HIV status disclosed to them. ART offered as first-line ART is a combination of tenofovir disoproxil fumarate (TDF), lamivudine (3TC), and dolutegravir (DTG). Depending on the existence of other comorbidities or treatment failure in the patient, other regimens are prescribed such as abacavir (ABC), azidothymidine (AZT), efavirenz (EFV), and ritonavir-boosted lopinavir or atazanavir (LPV/r or ATV/r).

### 2.2. Study Participants

AYALHIV aged 10–24 years on ART and registered at the HIV outpatient clinic were recruited. Any AYALHIV identified with either major psychotic disorder or history of organic brain disease such as stroke, tumors, and intracranial space-occupying lesions were excluded from the study. Participants' medical records were reviewed for clinical information for assessment of inclusion or exclusion in the study.

### 2.3. Sample Size and Sampling

The sample size was estimated to detect a prespecified width of a 95% confidence interval (95% CI) of 10% (± 5% precision) around a prevalence of depressive symptoms. Using a prevalence of 16.9% from a study in Nigeria by Adeyemo et al. [12], an estimated minimum sample size of 216 is needed, using the binomial exact (the Clopper–Pearson) CI calculation [13, 14]. The prevalence from Nigeria was selected due to similar geographical (West Africa) and cultural context as Ghana.

The study employed purposive sampling to recruit eligible AYALHIV into the study after establishing their eligibility from reviewing their medical records.

### 2.4. Data Collection and Tools

A structured questionnaire was used to obtain data on sociodemographic and clinical characteristics from study participants. Clinical characteristics obtained included HIV type, duration of HIV diagnosis, antiretroviral regimen, duration on ART, history of ever being lost to follow-up since treatment initiation (defined as a participant who has not been seen at the clinic for > 30 days since their last missed drug collection appointment), and viral load within the past one year. Information not obtained from participants was extracted and validated from patients' medical records.

The Patient Health Questionnaire for Adolescents (PHQ-A) comprising 13 items (the PHQ-9 and additional four questions/items) was used to screen for depression [15]. The PHQ-9 tool consists of nine items that measure the presence of depressive thoughts and feelings in individuals over the previous 2 weeks. The PHQ-9 is a four-point Likert scale with a range from 0 to 3, 0 for “not at all” responses and 3 for “nearly every day” responses. The total scores range from 0 to 27.

The HIV Stigma Scale 12 (HSS-12) was used to assess perceived stigma and discrimination among AYALHIV. It is a 12-item scale and a shorter version of the Berger HSS [16]. It comprises four domains, namely, “Personalized Stigma (3-item),” “Disclosure Concerns (3-item),” “Concerns about Public attitudes (3-item),” and “Negative Self-Image (3-item).” The responses to the 12 items are Likert scale rated as 1 “strongly disagree,” 2 “disagree,” 3 “agree,” and 4 “strongly agree.” Each item score ranges from 1 to 4 (3–12 for subscale), with higher scores reflecting a higher level of perceived HIV-related stigma. A total score ranging between 12 and 48 is obtained by summing all item scores.

The Brief Medication Questionnaire (BMQ) was used to screen for adherence and barriers to adherence among AYALHIV [17]. It is a self-report nine-item tool that includes a five-item regimen screen that assesses how patients take their medications over the past 1 week. It also includes a two-item belief screen that asks about drug effects and worrisome features and a two-item recall screen about potential difficulties remembering. Each question in each domain has a binary response (“0” for no and “1” for yes). The regimen screen domain was used in this study to assess adherence. For the regimen screen domain, a score of ≥ 1 indicates potential nonadherence.

The administered forms were reviewed daily to ensure completeness and to validate data.

### 2.5. Study Variables

The main outcome variable was the prevalence of depressive symptoms defined as at least a mild self-reported level of depression, which was defined as having a total PHQ-A score of ≥ 5 or dysthymia. This cutoff definition enables identification of all AYALHIV with depressive symptoms irrespective of the severity to offer the needed tailored intervention. For descriptive purposes, depressive scores were also subcategorized: scores of 5–9 indicated mild depression, 10–14 indicated moderate depression, and 15–27 indicated severe depression [15].

Dysthymia was defined as a “yes” response to one additional item “*In the past year have you felt depressed or sad most days, even if you felt okay sometimes?”* in the PHQ-A questionnaire [15].

All AYALHIV found to have severe depression were immediately linked to care to be evaluated and managed by the clinical psychologists and psychiatrists at the same hospital.

The independent variables included the sociodemographic and clinical characteristics and stigma. While there is a large variety of factors that we could have considered, we chose these three domains as the first two can be considered with a few well-defined variables and yet mechanistically be associated with a large number of potential risk factors for depressive symptoms, and stigma is described in the literature as a negative factor in mental health [18–20]. An adolescent was defined as any patient aged 10–19 years who accessed services at the HIV clinic [11]. A young adult was defined as any patient aged 20–24 years who accessed services at the AYALHIV clinic (WHO, 2019). Viral suppression was defined as having HIV viral load levels of ≤ 50 copies/mL, as outlined in the national HIV treatment guidelines [21].

An orphan was defined as a participant who had at least one parent dead. Having a history of *“Ever being lost to follow-up”* was defined as having any documentation in patients' clinical file of being lost to follow-up (not seen in care for at least 30 days) in care since being initiated on ART. *“Ever missed an appointment”* was defined as having any documentation in the participant's medical record of a missing scheduled clinical appointment since being initiated on ART.

### 2.6. Ethical Issues

Ethical approval was obtained from the Scientific and Technical Committee as well as the Institutional Review Board of the Korle Bu Teaching Hospital with approval identification number KBTH-STC/IRB/000128/2022. Permission was also obtained from the head of the HIV clinic, and written informed consent was obtained from participants aged ≥ 18 years. Assent was obtained from participants who were less than 18 years of age. Informed consent was also obtained from caregivers of participants who were less than 18 years. All data collected were treated confidentially by removing all personal identifiers from data collection tools and assigning unique study codes to each AYALHIV.

### 2.7. Data Analysis

Data were entered in an ongoing basis into Microsoft Excel 2019 and reviewed daily, thus there were no missing data. Data were then exported to Stata Statistical Software (StataCorp [2021]): Release 18. College Station, TX: StataCorp LLC, for analysis.

Categorical variables were summarized as frequencies and percentages. Continuous variables were summarized as medians with lower quartile (LQ) and upper quartile (UQ) or means with standard deviations. As tests for skewness in data are very sensitive to the sample size, we assessed the empirical distribution of continuous variables using visual aids of histograms and box plots. The prevalence of depressive symptoms was calculated by dividing the number of respondents with depressive symptoms by the total number of study participants and presented as a percentage. The surrounding 95% CI was calculated using the Clopper–Pearson exact method [13, 14]. Sociodemographic and clinical factors were cross-tabulated by presence and absence of the outcome of interest, and a *p* value from the chi-square test is reported for the omnibus test of independence for each factor (rows) and outcome levels (columns). Logistic regression was employed to determine the factors associated with the odds of depression with their corresponding odds ratio (OR) and 95% CI. For each factor of interest, we first estimated an unadjusted OR (95% CI), followed by an adjusted OR (95% CI), by keeping all of them in the adjusted logistic regression because our research question was concerned with all of them. In order to not overfit our multivariable logistic regression, we ensured that at least 5 cases (participants with depressive symptoms) were available for each category of the factor variable. For predictors that changed their association with the outcome from the unadjusted to adjusted analyses, we further investigated which other predictors possibly contributed to this phenomenon by examining their associations with each other using the chi-square test and ORs (95% CI). These data are presented in the Supporting Table 1. In such cases, since there is no statistical test to distinguish between a confounder and a mediator, we would need to consider a theoretical framework to have the basis for a mediation analysis, which was outside the scope of our research question and the cross-sectional nature of our study, but instead further addressed it in the discussion section. Due to the observational nature of this study, statistical power was not guaranteed for all statistical hypotheses tested in the logistic regressions (i.e., for all examined factors of interest) at the strict two-sided alpha level of 0.05, as it is not possible to plan the number of study participants in all categories of the variable combinations. Therefore, in order to minimize a potential Type II error, while balancing the Type I error, we relied on the magnitude of the effect size (OR) and the coverage of 95% CI including effects in mostly one direction to discuss important findings.

## 3. Results

### 3.1. Sociodemographic Characteristics

A total of 280 participants were enrolled in the study with 50.7% (142/280) being female. About 60% (169/280) of the participants were young adults, while the rest were adolescents (Table 1). The minimum and maximum ages of participants were 14 and 24 years, respectively, with a median age of 20 (IQR: 18–23) years. The majority (58.2% [163/280]) of participants had 7–12 years of formal education (Table 1). More than half (55% [150/280]) of the study participants were orphans (Table 1).

### 3.2. Clinical Characteristics and Perceived Stigma

The median duration of HIV diagnosis among participants was 13 years (LQ–UQ: 7.5–17 years). More than half (56.4% [158/280]) of participants had their duration of HIV diagnosis to be at least 10 years (Table 1). Most (99.6% [279/280]) participants had HIV Type 1 infection. All participants were receiving ART with a median duration of 12 years (LQ–UQ: 7–16). For the type of ART combination regimen, 98.9% (277/280) were on TDF/3TC/DTG with the rest on ABC/3TC/DTG. About 75% (211/280) of participants had HIV viral suppression. The mean overall stigma score was 29.4 (IQR: 25–34). The overall stigma scores ranged from 12 to 48.

### 3.3. Depressive Symptoms

The prevalence of depressive symptoms among AYALHIV was 40.7% (114/280) (95% CI: 34.9%–46.7%). Among those depressed, 56.1% (65/114) had mild depressive symptoms, 42.1% (35/114) had moderate depressive symptoms, and 1.8% (14/114) had severe depressive symptoms. More than half (53.6% [150/280]) of AYALHIV had dysthymia.

### 3.4. Factors Associated With Depressive Symptoms

In the unadjusted and adjusted analyses, being a female, an orphan, and higher perceived HIV stigma were associated with higher odds of reported depressive symptoms (Tables 1 and 2). Female respondents had 1.8 times higher odds of having depressive symptoms compared to their male counterparts (adjusted OR [aOR] = 1.8, 95% CI: 1.1–3.0). Participants who were orphans had 2.6 times higher odds of depressive symptoms compared to nonorphans (aOR = 2.6, 95% CI: 1.2–5.6). Participants with high perceived stigma had 2.2 times higher odds of having depressive symptoms (aOR = 2.2, 95% CI: 1.3–3.7).

Viral nonsuppression was associated with depressive symptoms but likely through its association with HIV stigma, as we found an unadjusted association between presence of viral nonsuppression and depressive symptoms (OR = 1.6, 95% CI: 0.95–2.8, Table 2), an association between viral nonsuppression and HIV stigma (OR = 1.7, 95% CI: 0.97–2.9, Supporting Table 1), while no association between viral nonsuppression and depressive symptoms in the adjusted model (Table 2). Age and educational level, as well as ever being lost to follow-up were not found to be associated with having depressive symptoms in either unadjusted or adjusted analyses (Table 2).

## 4. Discussion

The study assessed depressive symptoms' prevalence among AYALHIV and associated factors. Four out of every 10 AYALHIV were found to have depression with 88% having mild to moderate depression. Being female, being an orphan, and having high perceived HIV-related stigma were associated with having depressive symptoms. Viral nonsuppression was also associated with depressive symptoms, and in our study, this was in turn explained by its association with HIV stigma.

The prevalence of depressive symptoms among AYALHIV in our study was 41%. This was similar to findings of the prevalence of 46% reported amongst adolescents living with HIV in Uganda using the Center for Epidemiologic Studies' Depression (CES-D) Scale [22] and 44% in Botswana [23]. Our study findings also fit into the varying prevalence of depressive symptoms from 14% to 53% in SSA [7, 24, 25]. However, our finding was higher than a reported prevalence of 35.5%, 31%, 16%, and 18.1% among adolescents living with HIV in Ethiopia using Becks Depression Inventory II (BDI-II) [26]; Rwanda [27] and Tanzania [28] using PHQ-A; and Malawi using BDI-II [29], respectively. The differences in the depressive prevalence between our study findings and the others may be a result of the use of different tools and definitions in evaluating depressive symptoms. The difference may also be due to differences in geographical settings where psychosocial issues may also be dissimilar. For example, in Eastern and Southern Africa, HIV stigmatization is less pervasive [28, 29] compared to Ghana, where HIV is seen as a taboo. Our study's estimate of the prevalence of depressive symptoms was also higher than the global depression point prevalence of 34% and dysthymia of 4% among adolescents in the general population from 2001 to 2020 [30]. This can be attributed to neurobiological effects of HIV [31], ART side effects [32], as well as HIV stigma and social discrimination [33].

Females had almost twice the odds of males to report depressive symptoms in our study. This was similar to findings from a systematic review in 2021 by Ayano et al., [33] and other studies [29, 30], where being female was associated with having depressive symptoms. This is in agreement with reported depressive symptoms in the general population, refugees and asylum seekers, where females had higher rates of depressive symptoms compared to males [34, 35]. However, sex was not associated with having depressive symptoms among adolescents living with HIV in Uganda [22]. The possible reasons for the sex differences may be due to varying experiences in childhood and adolescence and differences in coping skills [36, 37]. In addition, due to the social and economic challenges faced by participants due to their orphan and caregiver type, females may face a higher risk of intimate partner violence, discrimination, social prejudice, and sexual and reproductive health concerns resulting in psychological stress compared to males [38]. Thus, adolescent girls and young women should be prioritized in HIV-mental health intervention development to address their increased risk of having depressive symptoms.

Participants who were orphans had three times the odds of having depressive symptoms compared to nonorphans. This was similar to findings in a systematic review, and another study in Rwanda, where being an orphan was also associated with having depressive symptoms [27, 39]. In contrast, a study conducted in Uganda reported that being an orphan was not associated with depressive symptoms [22]. In observational studies, the distribution of variables (e.g., social support, economic hardship, and caregiver characteristics) involved in mechanisms that can mediate the relationship between being an orphan and having depressive symptoms can vary from study to study, resulting in contradictory findings. The majority of our study participants who were depressed did not have both parents as caregivers, which can lead to poor social support and financial challenges [40]. Losing one or both parents is a difficult hurdle to cross for these AYALHIV, as they have to seek support from other caregivers or fend for themselves with their attendant problems, which can culminate in depression [27, 41].

Having high perceived HIV-related stigma was associated with having depressive symptoms in our study. This finding was similar to a study by Abebe et al. in Ethiopia, where stigma was associated with twice the odds of having depressive symptoms among adolescents living with HIV [26]. Since all our study participants had their status disclosed to them, and about 86% of them have been living with HIV for at least 5 years, they may have had experiences observing infected parents and colleagues being stigmatized [42]. In turn, they may have developed heightened perceived HIV-related stigma, leading to their withdrawal from social situations [40, 42, 43], and poor self-esteem [44], which can culminate in depression. Much is still needed to be performed in terms of counseling to reduce stigma, especially internalized stigma among the AYALHIV population [45].

Our study also showed that viral nonsuppression is likely associated with depressive symptoms through its association with HIV stigma. The existence of HIV-related stigma continues to disrupt HIV care and adversely impacts mental health during youth development [43]. Other studies have reported HIV-related stigma as a key barrier to engagement and retention in care, and adherence to ART, resulting in viral nonsuppression among the AYA population [18–20]. The potential negative impact of HIV-related stigma on adherence leading to viral nonsuppression increases the likelihood of onward transmission of HIV to others in the community [18, 19]. Therefore, further research is needed to examine how HIV stigma may mediate the association between viral nonsuppression and depressive symptoms among AYALHIV, or whether HIV stigma acts directly on depressive symptoms and indirectly through viral nonsuppression.

Increasing age or older age was not a significant factor for having depressive symptoms in our study. This was in contrast with findings from other studies including systematic reviews where older age was significantly associated with depression [22, 29–33, 39]. Adolescents living with HIV tend to get a better understanding and conceptualization of the HIV infection with increasing age [7]. This results in many unanswered questions among these adolescents that may account for increasing depression rates among them [7]. However, readily available counseling services for AYALHIV at the site for our study may have helped with understanding and coping among our older AYAs.

The study had some limitations. The study was cross-sectional, and therefore, we cannot comment on causality based on the associations found in this study. Furthermore, the cross-sectional nature of our study prevented us from formally examining a possible mediation at play in the triangle between HIV stigma, viral nonsuppression, and depressive symptoms. Participants were screened at a point in time, and so the onset or timing of depressive symptoms could not be established. Recall bias could have interfered with responses to questions framed to evaluate depressive symptoms in the previous 2 weeks. This study is from a single center and may not necessarily be representative of the broader AYALHIV population in Ghana, particularly those in rural areas or without access to specialized HIV care. In addition, the exclusion of participants with major psychotic disorders or organic brain diseases may lead to an underestimation of depression prevalence, as individuals with severe mental health conditions are systematically omitted.

Despite these limitations, the study establishes findings specific to the geographical setting which could be relevant to efforts at improving and integrating mental health into HIV care in Ghana. This study is also one of the few studies that has explored depression in AYALHIV in West Africa and adds to the body of evidence in the literature.

## 5. Conclusion

The prevalence of depressive symptoms in our study was high. The high rate of depression was associated with being female, an orphan, and having high perceived stigma. Routine monitoring and screening for depressive symptoms among AYALHIV, especially females and those who are orphans, is important for early intervention.

## Figures and Tables

**Table 1:** Sociodemographic and clinical characteristics of study participants at the Korle Bu Teaching Hospital stratified by depressive symptoms in Accra, Ghana, 2024.

Characteristic	Having depressive symptoms *N* (%)^#^	No depressive symptoms *N* (%)^#^	Total *N* (%)^$^	*p* value
Sex				0.01
Female	69 (48.6)	73 (51.4)	142 (50.7)	
Male	45 (32.6)	93 (67.4)	138 (49.3)	
Age category				0.65
Adolescents	47 (42.3)	64 (57.7)	111 (39.6)	
Young adults	67 (39.6)	102 (60.4)	169 (60.4)	
Years of formal education				0.81
0	3 (60.0)	2 (40.0)	5 (1.8)	
1–6	22 (43.1)	29 (56.9)	51 (18.2)	
7–12	65 (39.6)	99 (60.4)	164 (58.6)	
> 12	24 (40.0)	36 (60.0)	60 (21.4)	
Caregiver				0.01
Both parents	21 (27.6)	55 (72.4)	76 (27.1)	
Nuclear family member	44 (38.9)	69 (61.1)	113 (40.4)	
Extended family member	36 (52.9)	32 (47.1)	68 (24.3)	
Others^∗^	13 (56.5)	10 (43.5)	23 (8.2)	
Orphan				< 0.001
Yes	78 (51.0)	75 (49.0)	153 (54.6)	
No	36 (28.3)	91 (71.7)	127 (45.4)	
Duration of HIV diagnosis				0.86
< 5 years	14 (36.8)	24 (63.2)	38 (13.6)	
5–9 years	25 (42.4)	34 (57.6)	59 (21.1)	
≥ 10 years	75 (41.0)	108 (59.0)	183 (65.3)	
HIV type				0.41
Type 1	114 (40.9)	165 (59.1)	279 (99.6)	
Type 1 and Type 2	0 (0.0)	1 (100.0)	1 (0.4)	
Duration on ART				0.19
< 5 years	14 (29.2)	34 (70.8)	48 (17.1)	
5–9 years	33 (44.6)	41 (55.4)	74 (26.4)	
≥ 10 years	67 (42.4)	91 (57.6)	158 (56.4)	
Ever missed a clinical appointment				0.26
Yes	16 (50.0)	16 (50.0)	32 (11.4)	
No	98 (39.5)	150 (60.5)	248 (88.6)	
Ever been lost to follow-up				0.80
Yes	7 (22.6)	24 (77.4)	31 (11.1)	
No	107 (43.0)	142 (57.0)	249 (88.9)	
ART adherence				0.61
Yes	96 (41.0)	138 (59.0)	234 (83.6)	
No	18 (39.1)	28 (60.9)	46 (16.4)	
Viral load (cp/mL)				0.14
Suppressed (≤ 50)	79 (37.4)	132 (62.6)	211 (75.4)	
Nonsuppressed (> 50)	35 (50.7)	34 (49.3)	69 (24.6)	
Perceived stigma				0.02
High	68 (50.4)	67 (49.6)	135 (48.2)	
Low	46 (31.7)	99 (68.3)	145 (51.8)	

^∗^Others: Philanthropist, pastor, living on their own, partner.

^$^Column percent.

^#^Row percent.

**Table 2:** Associations of factors of interest with depressive symptoms among adolescents and young adults living with HIV at the Korle Bu Teaching Hospital in Accra, Ghana, 2024.

Characteristic	Depressive symptoms % (N)	Unadjusted OR^λ^ (95% CI)	*p* value	Adjusted OR^λ^ (95% CI)	*p* value
Sex					
Male	32.6 (45/138)	1		1	
Female	48.6 (69/142)	2.0 (1.2–3.2)	0.01	1.8 (1.1–3.0)	0.02
Age category					
Adolescent	42.3 (47/111)	1		1	
Young adult	39.6 (67/169)	0.9 (0.6–1.5)	0.65	0.6 (0.4–1.2)	0.14
Educational level					
Primary	43.1 (22/51)	1		1	
Secondary/vocational	39.6 (65/164)	0.8 (0.5–1.6)	0.66	1.2 (0.6–2.6)	0.64
Tertiary	40 (24/60)	0.9 (0.4–1.9)	0.74	1.4 (0.5–3.3)	0.52
Caregiver					
Both parents	27.6 (21/76)	1		1	
Nuclear family	38.9 (44/113)	1.7 (0.9–3.1)	0.11	0.9 (0.4–2.3)	0.90
Extended family	54.4 (37/68)	3.0 (1.5–5.9)	0.002	1.3 (0.5–3.5)	0.64
Others	52.2 (12/23)	3.4 (1.3–8.9)	0.01	2.5 (0.8–7.7)	0.11
Orphan status					
Not orphan	28.4 (36/127)	1		1	
Orphan	51.0 (78/153)	2.6 (1.6–4.3)	< 0.001	2.6 (1.2–5.6)	0.02
Ever been lost to follow-up					
No	43.0 (107/249)	1		1	
Yes	22.6 (7/31)	1.1 (0.4–3.2)	0.80	1.0 (0.3–2.8)	0.95
Viral load (cp/mL)					
Suppressed (≤ 50)	37.4 (79/211)	1		1	
Nonsuppressed (> 50)	50.7 (35/69)	1.6 (0.9–2.8)	0.10	1.4 (0.7–2.6)	0.35
HIV perceived stigma					
Low	31.7 (46/145)	1		1	
High	50.4 (68/135)	2.2 (1.3–3.6)	0.002	2.2 (1.3–3.7)	0.004

Abbreviation: 95% CI = 95% confidence interval.

^λ^OR = odds ratio.

## Data Availability

The data that support the findings of this study are available from the corresponding author upon reasonable request.

## Supporting Information

Supporting Table 1 shows the relationship between viral suppression and selected patient characteristics that had been reported to have an association with viral suppression.
