# Supplementary material for: Depressive Symptoms Among Adolescents and Young Adults Living With HIV on Antiretroviral Therapy: Outcomes From a Cross-Sectional Study in Accra, Ghana
Source: AIDS Res Treat. 2025 Aug 20;2025:3249809. doi: 10.1155/arat/3249809 (PMC12390515; doi:10.1155/arat/3249809)
Supplement: Supporting Information — Additional supporting information can be found online in the Supporting Information section. [file 3249809.f1.docx]

**Supplementary Table 1: Association between viral suppression and selected patient characteristics among adolescents and young adults living with HIV in Accra, Ghana, 2022.**

| **Variable** | **Viral suppression** | |  | **p-value** |
| --- | --- | --- | --- | --- |
|  | **Suppressed**  **n(%, 95% CI)** | **Unsuppressed**  **n(%, 95% CI)** | **OR (95% CI)** |  |
| **Age category** |  |  |  | 0.701 |
| Adolescents | 85 (76.6, 67.6-84.1) | 26 (23.4, 13.6-29.5) | Reference |  |
| Young Adults | 126 (74.6, 67.3-80.9) | 43 (25.4, 19.1-32.7) | 1.1 (0.6-2.0) |  |
| **Sex** |  |  |  | 0.404 |
| Female | 104 (73.2, 65.2-80.3) | 38 (26.8, 19.7-34.8) | Reference |  |
| Male | 107 (77.5, 69.7-84.2) | 31 (22.5, 15.8, 30.3) | 0.8 (0.5-1.4) |  |
| **Orphan Status** |  |  |  | 0.079 |
| No | 102 (80.3, 72.3-86.8) | 25 (19.7, 13.2-27.7) | Reference |  |
| Yes | 109 (71.2, 63.4-78.3) | 44 (28.8, 21.7-36.6) | 1.6 (0.95-2.9) |  |
| **Stigma** |  |  |  | 0.062 |
| Low | 116 (80.0, 72.6-86.2) | 29 (20.0, 13.8-27.4) | Reference |  |
| High | 95 (70.4, 61.9-77.9) | 40 (29.6, 22.1-38.1) | 1.7 (0.97-2.9) |  |

Abbreviation: 95%CI=95% Confidence Interval Based on Exact Binomial Method; OR=odds ratio for the odds of having unsuppressed HIV viral load compared to the reference group.
